# EZ-InSAR: An easy-to-use open-source toolbox for mapping ground surface deformation using satellite interferometric synthetic aperture radar

**DOI:** 10.1007/s12145-023-00973-1

**Published:** 2023-03-27

**Authors:** Alexis Hrysiewicz, Xiaowen Wang, Eoghan P. Holohan

**Affiliations:** 1 UCD School of Earth Sciences, University College Dublin, Dublin, Ireland; 2 SFI Research Centre in Applied Geosciences (iCRAG), University College Dublin, Dublin, Ireland; 3 Faculty of Geosciences and Environmental Engineering, Southwest Jiaotong University, Chengdu, China

**Keywords:** Easy-to-use InSAR toolbox, Ground deformation, Persistent scatterer, Small-baselines subset, Copernicus Sentinel-1

## Abstract

Satellite Interferometric Synthetic Aperture Radar (InSAR) is a space-borne geodetic technique that can map ground displacement at millimetre accuracy. Via the new era for InSAR applications provided by the Copernicus Sentinel-1 SAR satellites, several open-source software packages exist for processing SAR data. These packages enable one to obtain high-quality ground deformation maps, but still require a deep understanding of InSAR theory and the related computational tools, especially when dealing with a large stack of images. Here we present an open-source toolbox, EZ-InSAR (*easy-to-use InSAR)*, for a user-friendly implementation of InSAR displacement time series analysis with multi-temporal SAR images. EZ-InSAR integrates the three most popular and renowned open-source tools (i.e., ISCE, StaMPS, and MintPy), to generate interferograms and displacement time series by using these state-of-art algorithms within a seamless Graphical User Interface. EZ-InSAR reduces the user’s workload by automatically downloading the Sentinel-1 SAR imagery and the digital elevation model data for the user’s area of interest, and by streamlining preparation of input data stacks for the time series InSAR analysis. We illustrate the EZ-InSAR processing capabilities by mapping recent ground deformation at Campi Flegrei (> 100 mm·yr^−1^) and Long Valley (~ 10 mm·yr^−1^) calderas with both Persistent Scatterer InSAR and Small-Baseline Subset approaches. We also validate the test results by comparing the InSAR displacements with Global Navigation Satellite System measurements at those volcanoes. Our tests indicate that the EZ-InSAR toolbox provided here can serve as a valuable contribution to the community for ground deformation monitoring and geohazard evaluation, as well as for disseminating bespoke InSAR observations for all.

## Introduction

Interferometric Synthetic Aperture Radar (InSAR) is an important remote sensing technique for measuring ground surface motion from orbiting SAR satellites (e.g., Crosetto et al. 2016; Minh et al. 2020). SAR sensors emit and receive radar waves that can penetrate clouds, thus imaging during day or night and in all weather conditions. Space-based InSAR is widely used to monitor ground displacements related to earthquakes, volcanic eruptions, landslides, karstification, mining and groundwater abstraction (e.g., Biggs and Pritchard 2017; Boncori 2019; Hooper et al. 2012; Pinel et al. 2014; Sansosti et al. 2014).

InSAR works by exploiting the phase information of two radar images of a specific region collected at different times (Rosen et al. 2000). The images are co-registered, and the phase information is differenced to produce an interferogram. Once corrected for other contributions, such as topography and atmosphere, the interferogram represents a measure of ground motion (with respect to the satellite) that occurred between the image acquisition times. Given a stack of multiple SAR images acquired over weeks or years, ground deformation at millimetre scale can be mapped through time by using multi-temporal InSAR (MTI) techniques (Crosetto et al. 2016; Osmanoğlu et al. 2016). Established MTI approaches can be broadly classified into two categories: (1) Persistent Scatterer InSAR (PSI) and (2) Small Baseline Subset (SBAS) InSAR (Casu et al. 2006; Ferretti et al. 2001; Hooper 2008; Sadeghi et al. 2021; Shanker et al. 2011).

The availability of SAR satellite images free-of-charge has accelerated InSAR applications in recent years. The European Space Agency (ESA) launched the Sentinel-1A and Sentinel-1B SAR satellites in 2014 and 2016 for the European Union’s Copernicus Earth Observation program. These provide a minimum revisit frequency of 6–12 days, with all the data publicly accessible. A large amount of Sentinel-1 data has been archived for the continental regions of the Earth, which has proven to be a valuable data source for mapping ground deformation through time on national or even continental scales (Bischoff et al. 2020; Crosetto et al. 2020; Raspini et al. 2018).

InSAR data can be generated by several commercial or open-source software packages. Commercial codes include: ENVI SARscape (Sahraoui et al. 2006), SARproz (Perissin et al. 2011) and GAMMA software (Werner et al. 2000). Open-source software (Table 1) for generating interferograms include: the InSAR Scientific Computing Environment (ISCE) (Rosen et al. 2012); the InSAR processing system based on Generic Mapping Tools (GMTSAR) (Sandwell et al. 2011); the Sentinel Application Platform (SNAP) (Veci et al. 2014); the Stanford Method for Persistent Scatterers (StaMPS) (Hooper 2008; Hooper et al. 2004); the Python tool for estimating velocity and time-series from InSAR data (PyRate) (Wang et al. 2012); the Generic InSAR Analysis Toolbox (GIAnT) (Agram et al. 2013); and the Miami InSAR time-series software (MintPy) (Zhang et al. 2019).

**Table 1:** Existing popular open-source toolboxes for InSAR data processing and their main features. “Y” represents “Yes”, and “N” represents “No” for the presence of each feature. A toolbox available on Unix can, by definition, run on MacOS platform

Purpose	Software	Platform	Language	GUI	PSI	SBAS	ADF^2^
Interferometric processing	SNAP	Win/Unix/MacOS	Java	Y	N	N	DEM / Orbits
	ISCE2^1^	Unix (/MacOS)	C/C + + /Python/Shell Unix	N	N	N	DEM / Orbits
	GMTSAR^1^	Unix (/MacOS)	C/Shell Unix	N	N	Y	-
Displacement time series analysis	MintPy	Unix (/MacOS)/Windows	Python	N	N	Y	-
	StaMPS^1^	Unix (/MacOS)	MATLAB/Shell Unix	N	Y	Y	-
	PyRate	Unix (/MacOS)	Python	N	N	Y	-
	GIAnT	Unix (/MacOS)	Python	N	N	Y	-

^1^the software must be compiled by user

^2^Automatic Download Facilities (ADF)

Open-source software packages for MTI processing currently have three main disadvantages. First, nearly all are distributed for Linux or Unix operating systems with all parameters and functions controlled by command-line. Users thus need to be highly familiar with the technicalities of those operating systems. Only the SNAP software released by ESA has a GUI. Secondly, none facilitate automatic download of SAR data plus ancillary data such as DEMs and orbit files from online repositories. With more open-access data routinely acquired in the future from Sentinel-1 and other missions such as NiSAR (expected launch in 2023) tools for easy definition of an area of interest, for data accessibility checking and automated data download functionality are key. Thirdly, the open-source software packages are designed for dealing with only one sub-task of MTI analysis—i.e., for generating interferograms or for generating time-series of displacement. Users must switch between the different packages to complete an MTI analysis, which slows down the processing efficiency.

Here we present EZ-InSAR, a MATLAB-based toolbox that enables a first complete open-source InSAR processing chain from automatically downloading Sentinel-1 SAR images for the user’s area of interest to generating high-quality maps and time series of ground displacement. EZ-InSAR integrates existing open-source codes (ISCE, StaMPS, and MintPy), new data management tools and a GUI for intuitive and seamless InSAR processing with both PSI and SBAS approaches. In this article, we give a short description of InSAR processing theory in “Background to InSAR processing”. We present the main features of EZ-InSAR in “Design and implementation” and demonstrate its application to two active volcanoes (Campi Flegrei, Italy, and Long Valley, USA) in “EZ-InSAR toolbox application”. Finally, we outline future developments of EZ-InSAR in “Discussion and future development of EZ-InSAR”.

## Background to InSAR processing 

### Multi-temporal InSAR (MTI)

**Fig. 1:**
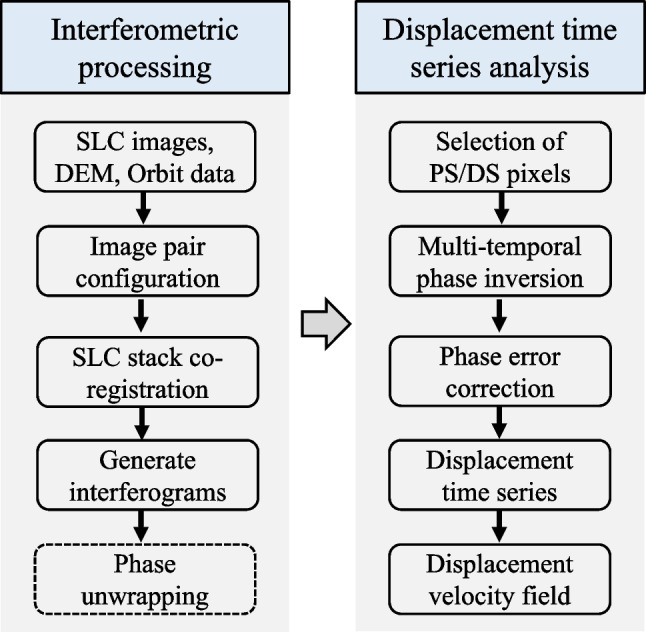
A generalised processing chain for retrieving ground displacement velocity and time series data from SAR imagery by multi-temporal InSAR analysis

The processing chain of MTI analysis can be divided into two workflows: (1) the generation of differential interferograms and (2) the generation of displacement time series (Fig. 1). Most modern satellite SAR missions, e.g., Sentinel-1, provide data that is pre-processed from unfocused raw data (Level 0) to a focused Single Look Complex image (SLC, Level 1), in which each pixel contains both amplitude and phase of the backscattered radar signal. The interferometric phase resulting from the differencing of the phase information in two SLC images is a lumped sum of contributions from ground deformation, SAR imaging geometry, surface topography, atmospheric delay, and InSAR decorrelation noise (Rosen et al. 2000).

The first workflow starts with the download of SLC and orbit data. This is followed by the co-registration of the SLC stack, image pair configuration, interferogram generation, and phase unwrapping (conversion of phase to displacement). The SAR satellite's orbital parameters and an external digital elevation model (DEM) are used to compute the phase components in an interferogram that are related to the SAR imaging geometry and the surface topography. These phase components can then be subtracted from the interferometric phase to produce the differential phase, which ideally represents the ground displacement between each SLC acquisition. However, other non-displacement components of the differential phase can include atmospheric delay, as well as residual errors from inaccurate orbital parameters and DEM data. Therefore, an important goal of MTI analysis is to isolate the deformation signal from the remaining error phases and atmospheric delays by analysing a stack of differential phase observations (Osmanoğlu et al. 2016).

The second workflow starts with selection of pixels (i.e., persistent scatterers and distributed scatterers) with high phase quality for displacement time series analysis. This is usually done by statistically analysing the temporal stability of SAR backscatter intensity and/or interferometric phase, or by using an a priori model (e.g., linearity) of the temporal evolution of displacements. Therefore, depending on the rules for defining them (Osmanoğlu et al. 2016), high-quality pixels may be those that in time display a relatively high and stable backscatter intensity or a relatively consistent to gradually changing phase. Using the differential phase of the selected pixels as observations, we can then construct an inversion model to obtain relative phase changes (i.e., displacements) for each SAR acquisition with respect to the reference epoch and a reference point. The phase inversion step also accounts for the correction of errors in the interferometric phase (i.e., phase contributions unrelated to ground motion), although the correction strategies differ for different MTI methods. For example, the atmospheric phase error can be estimated either by spatio-temporal filtering of the interferometric phase, or by using an external weather model or by using an a priori model of the temporal evolution of displacements (Agram et al. 2013; Li et al. 2022; Werner et al. 2003). Note that phase unwrapping in the first workflow can be bypassed for some MTI methods that use the differential phase between the neighbouring pixels as observations in the inversion model by assuming no phase ambiguity exists between them (Zhang et al. 2012).

PSI and SBAS are the two main types of MTI approaches. Differences between them include: (1) the method of forming InSAR image pairs, (2) the criteria of selecting high-quality pixels, and (3) the phase inversion model. The SAR image pairs in the PSI approach are formed by using a single reference scene; those in the SBAS approach are formed by using multiple reference scenes. The PSI technique selects pixels representing scatterers on the ground that persistently yield high amplitude and/or low phase variance in those pixels. The SBAS method deals with pixels representing more distributed and/or lower amplitude scatterers on the ground by using interferometric coherence as a selection criterion. The interferometric coherence is the correlation between two SAR images: as such the coherence is a direct estimate for the accuracy of the determination of the interferometric phase (Zebker and Villasenor 1992). Additionally, the SBAS approach generally involves multi-looking (i.e., pixel averaging) on the interferograms to reduce noise levels, although it is not mandatory, and it can be replaced or augmented by spatial or temporal filtering. For the phase inversion model, PSI approaches commonly use assumptions about the temporal change of ground deformation (e.g., linear, or seasonally oscillating), although some modified PSI techniques, such as the StaMPS method (Hooper et al. 2004), do not need such assumptions. In the SBAS approach, the greater number of redundant interferometric observations arising from the multi-reference method of forming image pairs helps to constrain the phase inversion model without needing an a priori assumption about the displacement behaviour in time. Both have advantages (and disadvantages) that depend largely on the target area’s spatiotemporal features. Generally, PSI is suitable for areas with localised, high-reflectivity radar back-scatterers, such as urban regions and man-made infrastructure. SBAS is suitable for areas with distributed, less reflective back-scatterers, such as in rural regions.

### Satellite acquisition modes

The main imaging mode of Sentinel-1 is the Interferometric Wide Swath Mode (IW). This uses a progressive scan (TOPS) antenna beam steering technique to provide a 250 km wide image swath consisting of three sub-swaths at c. 3 m × 22 m pixel size (Potin et al. 2019). Each swath consisting of several bursts (Torres et al. 2012). ESA released the Sentinel-1 TOPS SAR data in the format of SLC (L1), a standard format that is for interferometric processing, which can be fed into a standard InSAR processing chain after co-registration. Because of variable azimuth spectral properties of TOPS SAR data, an Enhanced Spectral Diversity (ESD) method using the phase of the burst overlay region of Sentinel-1 SAR data is usually employed to make sure a co-registration error smaller than 1/100 pixel (Yague-Martinez et al. 2016). The ESD assisted co-registration method is now supported by nearly all the popular InSAR processing tools, which will be introduced in the next section. Several international and national agencies, such as ESA and the Alaska Satellite Facility (ASF), support the distribution of Sentinel-1 data through web Application Programming Interface (API) services, which make it feasible to develop an automatic processing chain.

Sentinel-1 satellites also operate in Stripmap mode, which is the conventional acquisition mode for most SAR sensors. This mode can be less challenging as the co-registration accuracy for obtaining a suitable interferometry image at a spatial resolution of several metres can be < 1/10 pixel. However, the swath width is 80 km for the Sentinel-1 Stripmap mode, compared to 250 km for the IW mode. Current X-band satellites, such as COSMO-SkyMed, TerraSAR-X and PAZ, also operate in Stripmap-like mode.

## Design and implementation

### Toolbox structure and user interface

**Fig. 2:**
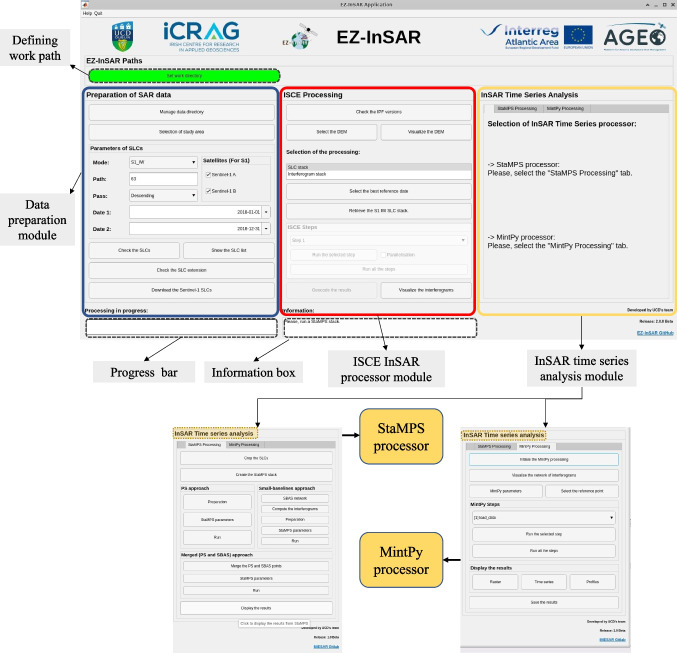
A snapshot of the interface of EZ-InSAR

EZ-InSAR consists of three modules: (1) *Preparation of SAR Data*, (2) *ISCE Processing*, and (3) *Time Series Analysis*. Each module is accessed through one of three main panels in the user interface (Fig. 2). A button to define an EZ-InSAR working directory path is on the top left of the interface. At the base of the GUI, there is a bar showing the progress of each InSAR processing step and an information box showing the progress state and additional useful information (i.e., errors) during data processing.

**Fig. 3:**
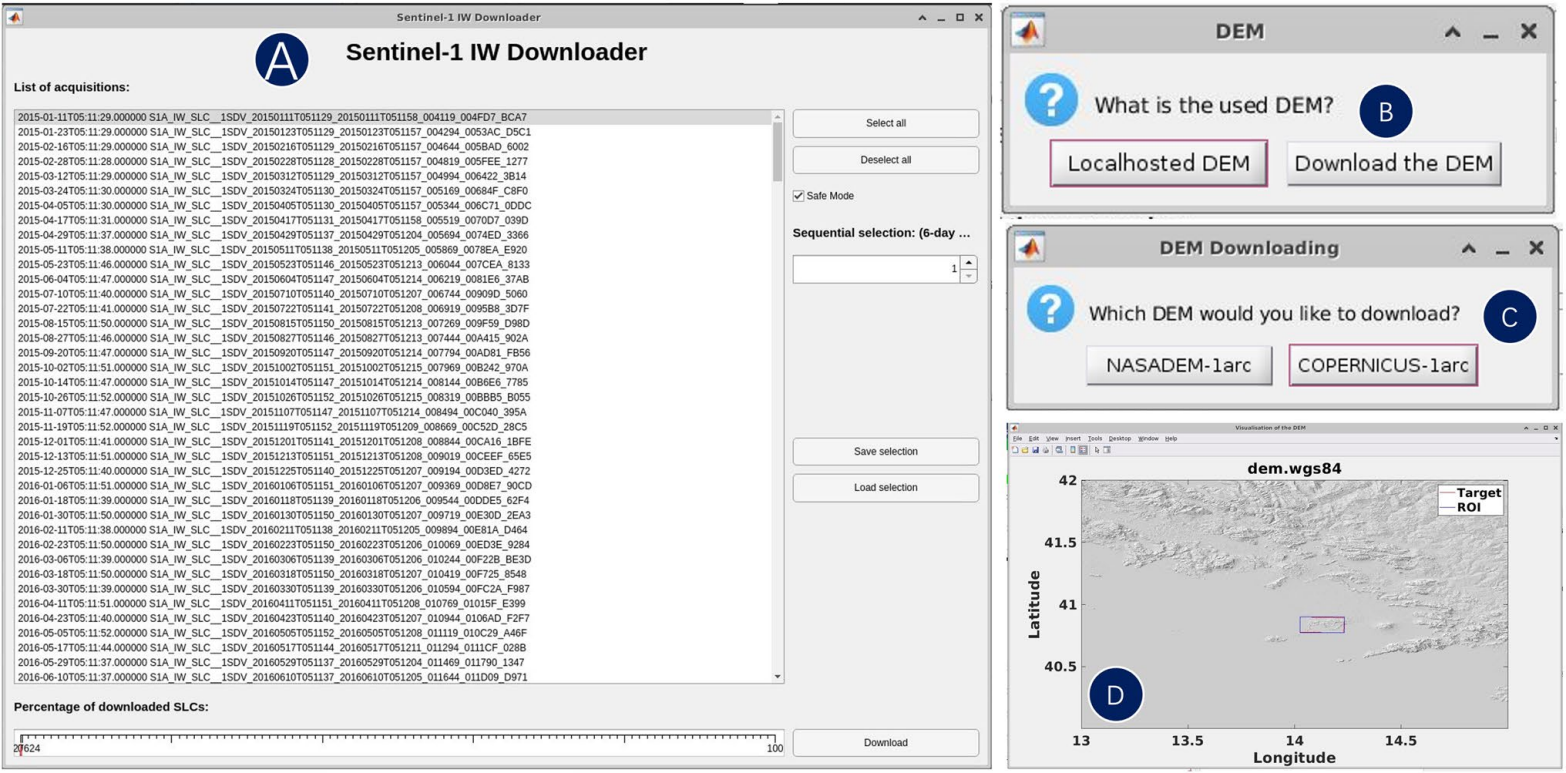
The interfaces for downloading the Sentinel-1 data and preparing the DEM for InSAR processing. (**A**) Windows for downloading the Sentinel-1 data associated with the selection options. (**B**) The dialog of options for selecting the local existing DEM or downloading a new DEM. (**C**) The dialog of options for downloading either NASA DEM or Copernicus DEM. (**D**) The visualization tool for displaying the shade relief map of DEM

The *Preparation of SAR Data* module includes functions for searching and downloading Sentinel-1 SAR data. EZ-InSAR supports the use of a KML file exported from Google Earth to define a study region. Once the study region is determined, the API provided by the ASF is then used to search the archive of available Sentinel-1 SAR images based on the input filtering keywords, such as the path number, flight direction of satellite (i.e., ascending or descending), and the desired time span. EZ-InSAR can then download the SAR data automatically after confirming the returned data list (see Fig. 3). A DEM covering the study region is required before running interferometric processing. Here EZ-InSAR provides options of automatically downloading either the SRTM DEM (Farr et al. 2007) or the Copernicus DEM from Amazon Web Services (ESA and Sinergise 2021). Both DEMs have a spatial resolution of 30 m and are saved in GeoTIFF format. EZ-InSAR also supports the input of a third-party DEM in GeoTIFF format as specified by the user (See Fig. 3). To help check the quality and coverage of the DEM, we provide a display option allowing the user to visualize a shaded relief map of the downloaded DEM overlapping onto a geographic base map.

The *SAR Interferometry* module includes the functions for interferometric processing. We selected the ISCE package, initially developed by a team from NASA’s Jet Propulsion Laboratory and from Stanford University for this purpose (Rosen et al. 2012). ISCE is a highly hierarchical package with Shell and Python scripts to control the different applications based on specified tasks, such as the task of generating a stack of coregistered SLC images, a stack of interferograms without phase unwrapping, and a stack of unwrapped interferograms. EZ-InSAR here gives the options to generate an “SLC stack” or an “Unwrapped interferogram stack”, depending on whether the user wishes to run a StaMPS-based or a MintPy-based time series analysis, respectively. EZ-InSAR also allow bidirectional conversions between these two data stacks (Fig. 11), so that one can more easily conduct and cross-check the MTI analysis with different approaches. EZ-InSAR supports the generation of the data stack either step-by-step or by batch processing in a parallelised computation.

The *Time Series Analysis* module provides the option of using either the StaMPS or the MintPy time-series processors. It also undertakes initial checks on input data suitability for these. The StaMPS package is mainly written in MATLAB and can perform both PSI and SBAS analyses at full image resolution. StaMPS also enables integration of the PSI and SBAS results to enhance the density of measurement points (Hooper 2008). MintPy is written in Python and provides several different SBAS processing algorithms for correcting interferometric artifacts (e.g., phase unwrapping error, tropospheric delay, and topographic residual) and for obtaining displacement time series (Zhang et al. 2019). The StaMPS and MintPy processors can be activated by clicking the corresponding tabs in the panel (Fig. 2). The GUI panels enable control of the parameters that will control the MTI analysis. Again, the time-series processing with either StaMPS or MintPy can be done either step-by-step or by batch processing.

**Fig. 4:**
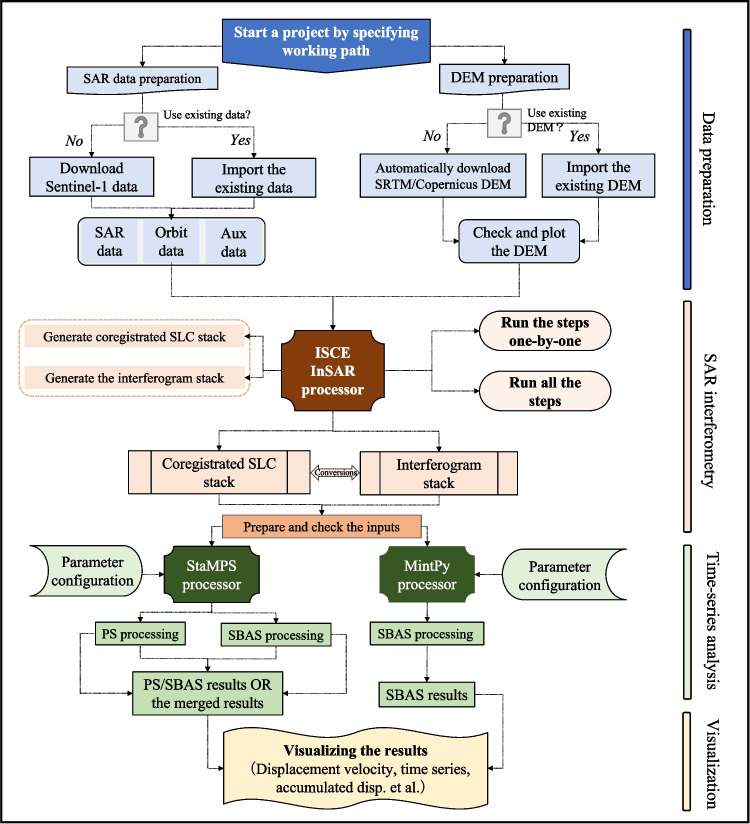
A flow chart of SAR data processing in EZ-InSAR

The location of the reference point for displacement has a great influence on the final InSAR results. For example, EZ-InSAR allows the users to select the reference point, for MintPy-based processing, interactively on based on optical satellite images provided by MATLAB and hosted by Esri. Finally, users can export the velocity field and the displacement time series from EZ-InSAR into several formats, such as GeoTIFF, KMZ, and QGIS, such that they can be further exploited by the other software for visualization and analysis. Figure 4 shows a flow chart of functionality and data processing within each of these modules, which collectively facilitate a full MTI processing chain.

### Additional tools to assist MTI in EZ-InSAR

**Fig. 5:**
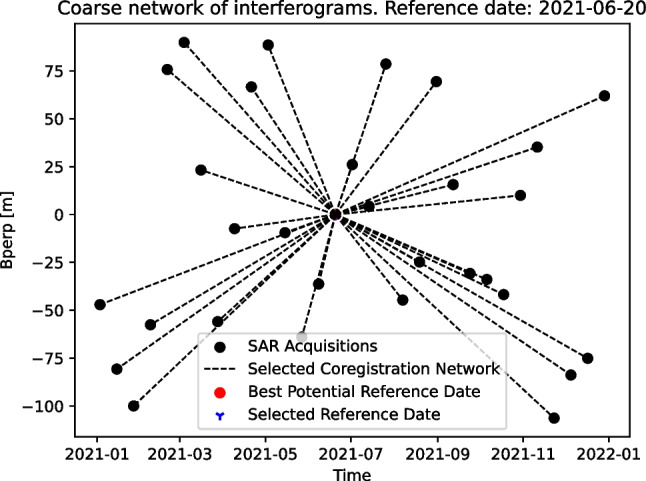
The tool in EZ-InSAR for selecting the optimal reference image based on the scatter plots of the temporal and perpendicular baselines of SAR images

**Fig. 6:**
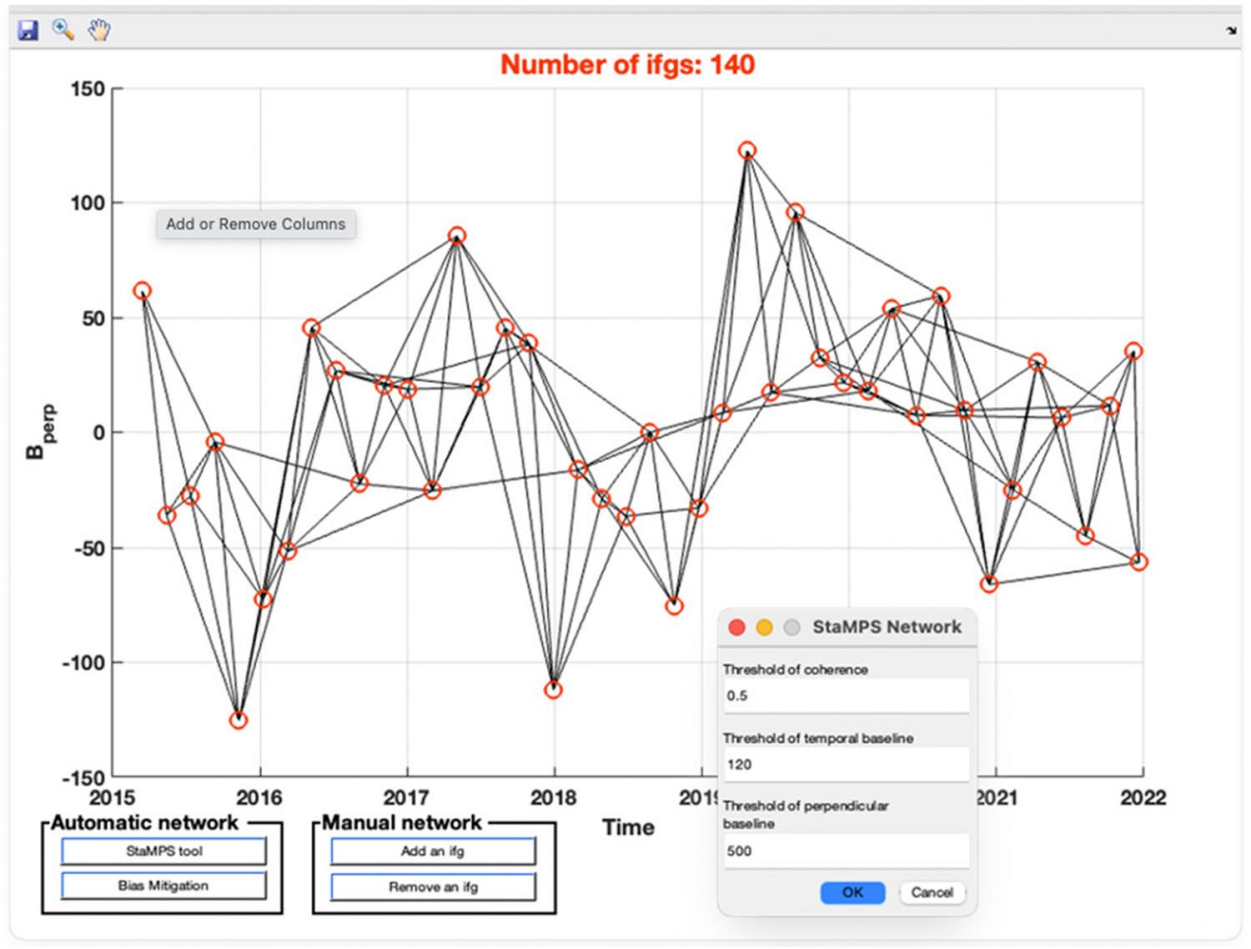
The tool in EZ-InSAR for generating and modifying the InSAR inversion network when running StaMPS. Users can use thresholds shown in the upper right figure to create the network, while they can also adjust the network by adding or deleting the desired image pair connection through the “Manual network tool” shown below the network

EZ-InSAR also has several unique tools to optimize the SAR data processing and to obtain high quality ground displacement measurements. Firstly, we designed a tool to automatically select the SAR acquisition in the geometric centre of the perpendicular baseline vs temporal baseline network as the optimal reference image (Fig. 5). Coregistration accuracies between the reference and secondary SAR images should thereby be improved since the temporal and perpendicular baselines of image pairs greatly influence the interferometric coherence. Secondly, we developed a display window allowing users to easily visualize all SAR interferograms, unwrapped phase maps, and coherence maps within a stack. Using this tool, the user can inspect the quality of the interferograms and drop bad ones from the MTI analysis (Fig. 12). Thirdly, we developed a tool that allows the user to manually modify the SAR image connection network by removing or adding specific image pairs in the MTI analysis (Fig. 6). For example, if the initial MTI analysis has revealed that some images are strongly contaminated by atmospheric delay, because they exhibit large residuals in the initially retrieved displacement time series, then we can drop these un-desired image pairs and re-run the MTI analysis to improve the results.

## EZ-InSAR toolbox application

### Data processing for Campi Flegrei and Long Valley calderas

**Fig. 7:**
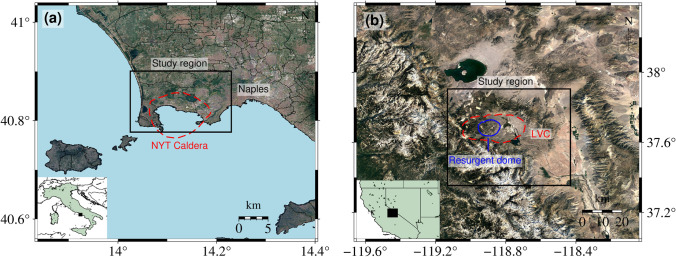
Satellite maps, provided by Google Earth, of the test sites Campi Flegrei volcano (**a**) and Long Valley caldera (**b**). The rectangles are the coverage of the Regions of Interest. The red dashed lines are the NYT caldera boundary in (**a**) and LVC boundary in (**b**). The solid blue line within LVC outlines the Resurgent Dome

Campi Flegrei Caldera, Italy, is a 13-km-wide depression that hosts a set of young volcanic vents and active geothermal areas. It is partly submerged beneath the Gulf of Pozzuoli, and it is highly urbanised, encompassing part of the city of Naples (Pepe et al. 2019) (Fig. 7a**)**. The most recent eruption was from the Monte Nuovo vent in 1538 (Di Vito et al. 1987). Episodes of significant uplift and subsidence within caldera have occurred since Roman times, and inflation has occurred at an increasing rate since 2005 (De Martino et al. 2021). Here we can test EZ-InSAR’s performance in an urban to sub-urban area with large ground motion and medium/high InSAR coherence.

Long Valley Caldera, USA, is a 32 × 16 km depression that hosts a resurgent dome and several active geothermal areas (Fig. 7b**)**. It lies at the eastern edge of the Sierra Nevada mountains and occupies a mostly rural area with several small towns. Two younger volcanic systems on the margins of the caldera, Mammoth Mountain, and Mono-Inyo Craters, have most recently erupted about 600–700 years ago. Long Valley Caldera has exhibited relatively small-magnitude deformation related to hydrological forcing and volcanic activity since 1980s (Silverii et al. 2020, 2021). Here we can evaluate the performance of EZ-InSAR in revealing subtle ground deformation in a rural setting with lower InSAR coherence.

The black rectangles in Fig. 7a and b show the footprints of the KML files that we used in EZ-InSAR to download Sentinel-1 SAR images for the two test sites (Table 2**)**. We applied both the StaMPS-based PSI and MintPy-based SBAS approaches at the Campi Flegrei volcano (see parameter settings in Figs. 13 and 15). Given the dense vegetation (forests) there, we employed the SBAS approach in StaMPS and MintPy at Long Valley caldera (see parameter settings in Figs. 14 and 15). Note that we used the default threshold values of StaMPS to select the high-quality pixels. We adopted the elevation-phase correlation method to remove the atmospheric phase delay (Li et al. 2019), and we used a linear fitting model to deramp the velocity fields. Note that StaMPS provides options of implementing spatial and temporal filtering to the results, while MintPy does not. Unlike the data processing based on full resolution SLC data in StaMPS, MintPy works by exploiting the unwrapped phase of multi-looked interferograms. Multi-looking factors of 2 in SAR azimuth direction and 10 in range direction were used to generate the interferogram stacks.

**Table 2:** The Sentinel-1 SAR datasets used for the two test sites

	Time span	Number of images	Path number	Flight direction	Processing area (km^2^)	MTI method used
Campi Flegrei	2021/01/03 – 2021/12/29	31	22	Ascending	~ 240	StaMPS PSI MintPy SBAS
Long Valley caldera	2020/01/05 – 2021/12/25	41	144	Descending	~ 3720	StaMPS SBAS MintPy SBAS

The processing time is mainly related to the SAR data amount, the area coverage of the study region, the selected MTI approach. On our server, which possesses an AMD EPYC™ 7662 CPU with 64 cores (2.0 GHz, max. 3.3 GHz) and a RAM memory of 1 TB (2,933 MHz), for example, the total processing times were 4 and 6 h for the PSI and SBAS analyses for Campi Flegrei caldera, respectively; the SBAS analyses ran for a couple of days for Long Valley Caldera.

### Ground deformation at Campi Flegrei Caldera

**Fig. 8:**
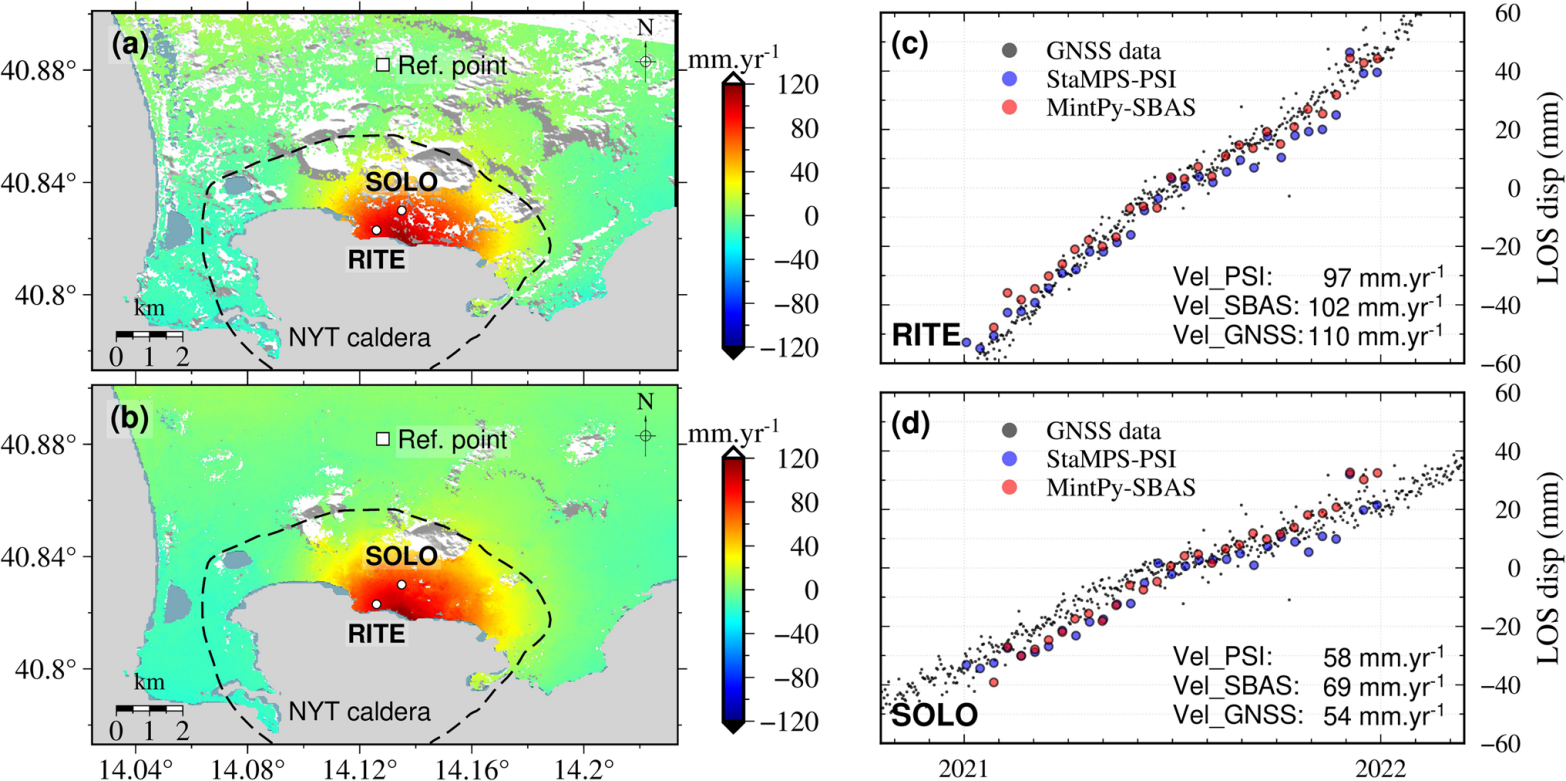
Multi-temporal InSAR results for the Campi Flegrei caldera from January to December 2021 inclusive. The mean ground displacement velocity maps derived from the StaMPS-based PSI and MintPy-based SBAS are in (**a**) and (**b**), respectively. The white circles represent the GNSS sites. The dashed outlines give the extent of the Neapolitan Yellow Tuff (NYT) caldera. The white square represents the reference point of the InSAR displacements. The comparisons between the GNSS and InSAR observations at the two GNSS sites RITE and SOLO are given in (**c**) and (**d**). The grey points represent the GNSS observations, while the blue and red circles represent the StaMPS-based PSI and MintPy-based SBAS measurements, respectively. The mean displacement velocities from the three kinds of observations are also annotated

Figure 8 shows the mean ground displacement velocities at Campi Flegrei caldera as mapped by using the StaMPS-based PSI and MintPy-based SBAS approaches, according to default parameters of processing. Both the velocity fields show surface uplift of up to ~ 120 mm·yr^−1^ along the satellite Line-of-Sight (LOS) direction in 2021. The main deforming region covers an area of about 18 km^2^. The two maps mainly differ in the number and spatial coverage of velocity measurements, which are greater with the SBAS method. The InSAR displacement time series agree well with GNSS observations at two local monitoring sites provided by De Martino et al. (2021): RITE and SOLO** (**Fig. 8c and d**)**. We converted the west–east, south-north, and vertical components of the GNSS displacement time series into a component in the satellite LOS direction. The calculated LOS displacement velocities based on the GNSS data are 110 and 54 mm·yr^−1^ at RITE and SOLO, respectively. The mean displacement velocities from the PSI and SBAS InSAR are 97 and 102 mm·yr^−1^ at the GNSS site RITE, and 58 and 69 mm·yr^−1^ at the GNSS site SOLO. The largest discrepancies are ~ 15 mm·yr^−1^. The RSME values between the GNSS data and InSAR displacements are 5.7 mm and 7.7 mm for the StaMPS-based PSI approach, 6.3 mm and 7.6 mm for the MintPy-based approach, at RITE and SOLO, respectively.

### Ground deformation at Long Valley Caldera

**Fig. 9:**
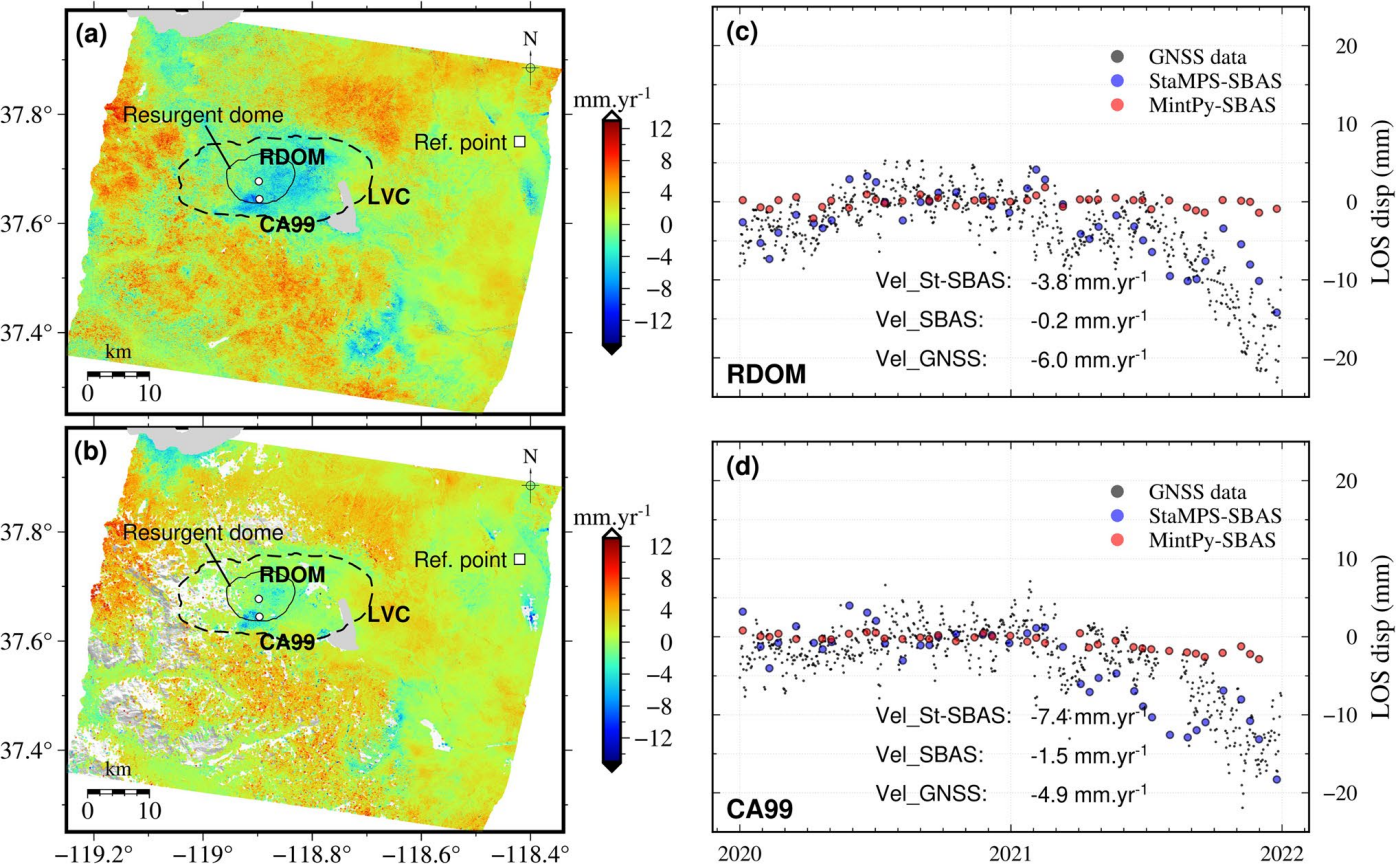
Multi-temporal InSAR results for Long Valley Caldera from 2020 to 2021 inclusive. The mean displacement velocity maps derived from the StaMPS-based SBAS and MintPy-based SBAS are in (**a**) and (**b**). The white circles represent the GNSS sites. The white square represents the reference point of the InSAR displacements. The comparisons between the GNSS and InSAR observations at the two GNSS sites RDOM and CA99 are given in (**c**) and (**d**). The grey points represent the GNSS observations, while the blue and red circles represent the StaMPS-based SBAS and MintPy-based SBAS measurements, respectively. The mean displacement velocities from the three kinds of observations are also annotated

Figure 9 shows the ground motion velocity maps derived for the Long Valley caldera from StaMPS-based SBAS and MintPy-based SBAS, respectively, according to default parameters of processing. The two SBAS approaches reveal local subsidence at a rate of about -15 mm·yr^−1^ near the south edge of the resurgent dome, where a geothermal plant (named Casa Diablo) is located. Previous InSAR investigations using ENVISAT SAR data from 2003 to 2010 have also revealed subsidence near this geothermal plant, indicating that the subsidence has persisted in recent decades (Hetland et al. 2012). To the east of the resurgent dome, we observe an apparent widespread LOS subsidence within the caldera (clearer in the StaMPS result).

Figure 9c and d show the displacement time series at the two continuous GNSS stations, RDOM in the centre of the resurgent dome and CA99 near the geothermal exploration site. The GNSS measurements, provided by the Nevada Geodetic Laboratory, were calibrated by using the P653 station. They show that both sites were relatively stable from January 2020 until January 2021 but subsided from January 2021 until December 2021. The LOS displacement velocities at RDOM and CA99 are -6.0 and -4.9 mm·yr^−1^, respectively, over the two years. The MintPy-based SBAS measurements are -0.2 and -1.5 mm·yr^−1^, which underestimate the GNSS-derived values by 5.8 and 3.4 mm·yr^−1^, respectively (i.e., by about 97% and 70%). The StaMPS-based SBAS measurements at the two stations are -3.8 and -7.4 mm·yr^−1^, which respectively overestimate and underestimate the GNSS velocity magnitudes by about 37% and 51%. However, the StaMPS-based approach successfully captures the temporal evolution of the deformation at Long Valley caldera, with initial stability in 2020 succeeded by subsidence at both sites in 2021. The RSME values between the GNSS data and InSAR displacements are 4.8 mm and 3.8 mm for the StaMPS-based SBAS approach, and 7.0 mm and 4.9 mm for the MintPy-based approach, at CA99 and RDOM, respectively.

## Discussion and future developments of EZ-InSAR

EZ-InSAR is a user-friendly solution for generating ground displacement fields from different MTI methods (StaMPS PSI, StaMPS SBAS and MintPy SBAS). The coherent workflow of EZ-InSAR allows the easy and efficient manipulation of the SAR data and their importation into the MTI processing chain. The tests of the toolbox on areas of contrasting land-cover characteristics and displacement rates, represented by Campi Flegrei and Long Valley calderas, shows that the ground displacement can be successfully extracted with either the PSI or SBAS techniques.

At Campi Flegrei caldera, the ground surface has uplifted at a velocity more than 100 mm·yr^−1^ along the LOS direction in 2021. Results from PSI (StaMPS) and SBAS (MintPy) approaches are similar to each other and to GNSS measurements on the ground. The largest velocity discrepancies between the InSAR and GNSS measurements reach about 10% of the velocity magnitude. Given the short duration of the time series, discrepancies between GNSS and InSAR measurements are normal. They could be related to atmospheric delay errors and topographic errors in the InSAR processing.

At Long Valley Caldera, local LOS subsidence was mapped by SBAS techniques. The maximum LOS subsidence rate from InSAR was about 15 mm·yr^−1^ during 2020–2022, which is about one order magnitude smaller than observed at the Campi Flegrei volcano. The highest velocity discrepancies between the SBAS (MintPy) and GNSS reach about more than 90% of the GNSS velocity magnitude. The discrepancies between the InSAR and GNSS measurements in this case indicate that the MTI analysis results should be carefully interpreted of when dealing with study sites with subtle and/or non-linear ground deformation. Again, a longer time of observations may be needed to correct for topographic, seasonal atmospheric effects and potential bias more successfully (e.g., Ansari et al. 2021). However, the temporal behaviours of displacements from StaMPS-based SBAS and from GNSS results are similar (slow uplift or stable in 2020, then slow subsidence in 2021).

We have created a GitHub repository (https://github.com/alexisInSAR/EZ-InSAR) for sharing the EZ-InSAR toolbox. Here the user can find full documentation of EZ-InSAR, with installation instructions and some examples of processing applications. Currently, the version of EZ-InSAR (2.0.0 Beta) also implements the processing of some Stripmap-like data, Sentinel-1 IW, and gives instructions to add new sensors. The Discussion panel of the GitHub repository is available for discussion and interactions with users. Our aims with this repository are to make it possible to rapidly disseminate our future updates of EZ-InSAR and to enhance the toolbox functions from community-driven contributions.

**Fig. 10:**
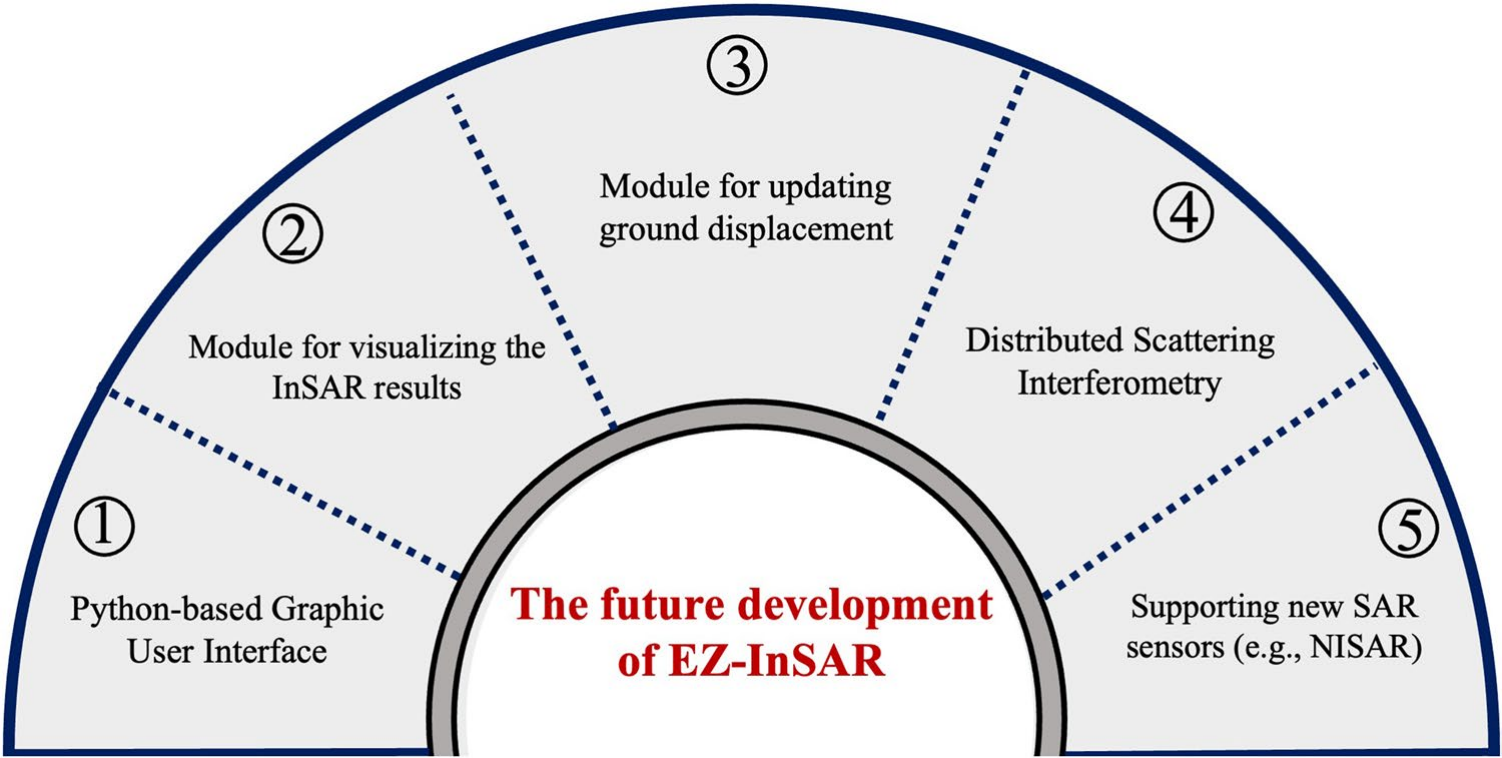
A sketch map summarizing the future priority development plans of EZ-InSAR

**Fig. 11:**
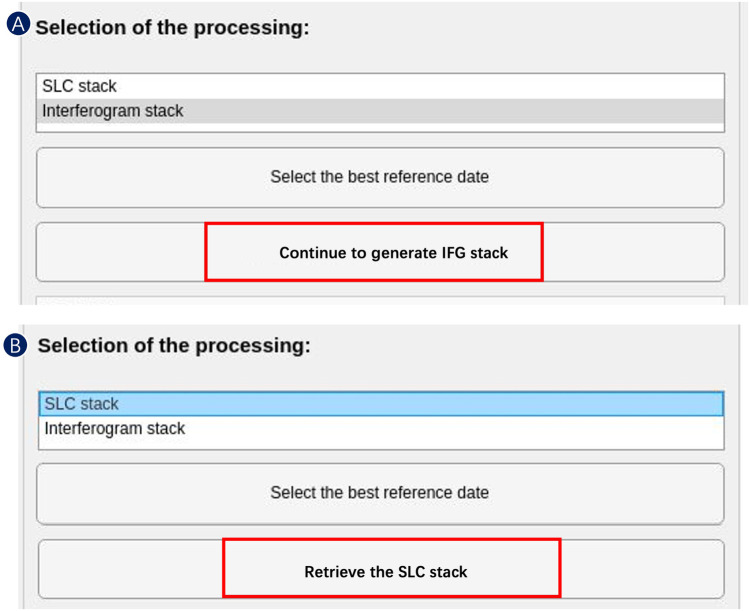
The interfaces for running conversions between the interferogram stack and SLC stack generated from ISCE

**Fig. 12:**
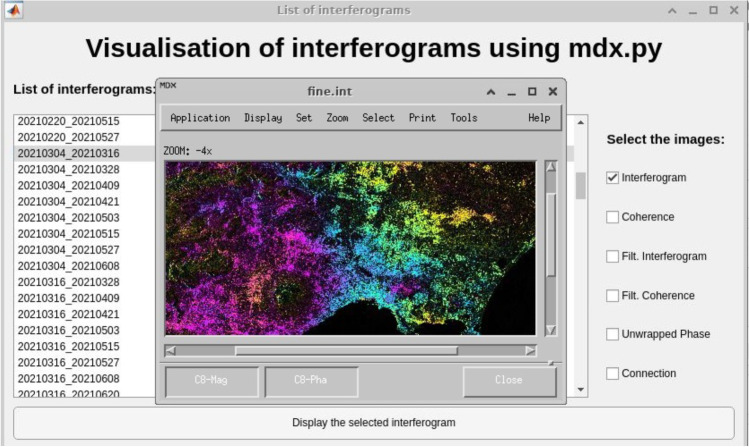
The toolkit for displaying the InSAR results generated from ISCE

**Fig. 13:**
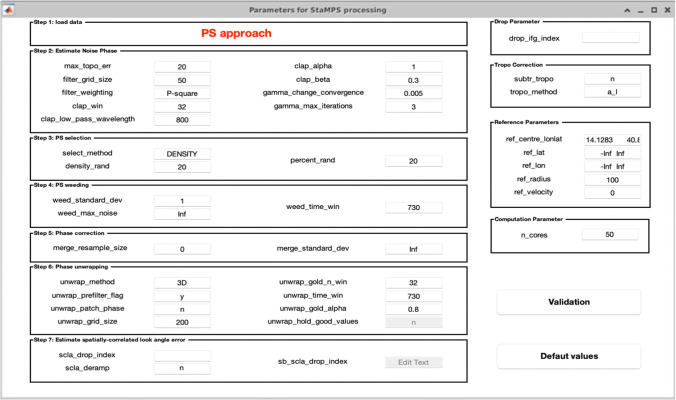
The parameter settings for running the StaMPS PSI approach at the test site of Campi Flegrei volcano. The amplitude dispersion index was 0.4

**Fig. 14:**
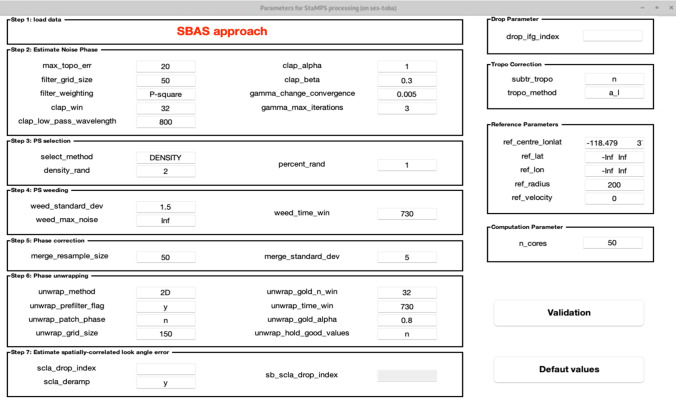
The parameter settings for running the StaMPS SBAS approach at the test site of Long Valley Caldera. The amplitude dispersion index was 0.6

**Fig. 15:**
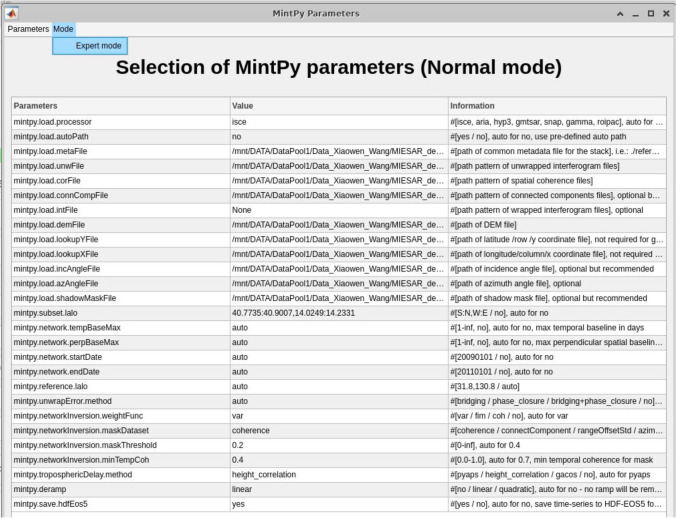
The parameter settings for running the MintPy SBAS approach at the two test sites

Considering the increasing accessibility of space-based SAR data in future and the recent developments of MTI analysis techniques, we here give an outlook for the future development of EZ-InSAR toolbox (Fig. 10).

While EZ-InSAR is currently developed in MATLAB language, our priority is to translate the set of EZ-InSAR scripts to Python language. By providing the option to avoid requiring a commercial language to run the software, the dissemination of EZ-InSAR should be made easier. During this translation, we will focus on the cross-platform compatibility to make EZ-InSAR available for most operating systems. However, the MATLAB language will likely be required to use StaMPS.

We will enhance the visualization and plotting capabilities of EZ-InSAR in the future releases, so that users can directly save high quality and publication-ready images.

EZ-InSAR currently processes an entire SAR data stack from start to end. Ideally, the displacement time-series and velocity maps could be updated with newer SAR acquisitions without reprocessing the entire stack. Timely updating of ground displacement is critical when applying MTI techniques to near real-time monitoring of geohazards. Therefore, EZ-InSAR should in future be capable of updating the ground displacements by using only the new acquisition and the previously adjacent SAR images.

More recently developed MTI techniques have made significant advances in increasing the density and quality of ground deformation measurements by exploring the interferometric phase of statistically homogeneous pixels surrounding the PSI pixels. These techniques can be grouped as Distributed Scattering Interferometry (DSI) approaches (Even and Schulz 2018; Ferretti et al. 2011). DSI aims to reduce the interferometric phase noise by using phase linking or phase triangulation algorithms based on full network of SAR interferograms. More recent open-source packages available for displacement time series analysis, such as the FRInGE (Fine Resolution InSAR using Generalized Eigenvectors) (Fattahi et al. 2019) and MiaplPy (MIAmi Phase Linking in Python) packages (Mirzaee et al. 2019) can be incorporated into EZ-InSAR in the future.

In addition, several new SAR satellite missions will commence in the next few years. For example, ESA plans to launch the Sentinel-1C/D SAR satellites in 2023 (Spataro et al. 2021). Also, a dual-frequency L- and S-band SAR satellite, NASA-ISRO SAR (NISAR), will be launched in 2023 (Rosen et al. 2015). All these satellites will have a free data policy. Therefore, we will extend the functions of EZ-InSAR to support the searching, downloading, and processing of the SAR data of these new satellites.

Finally, other new functionalities can be added: for example, the implementation of SNAP software instead of ISCE package for InSAR processing (Veci et al. 2014); support of other SAR data currently in orbit (e.g., RADARSAT-2); or automatic selection of Sentinel-1 images according to the Region of Interest. 

## Summary

In this paper, we present an open-source toolbox named EZ-InSAR for mapping ground deformation by using the multi-temporal InSAR (MTI) analysis technique. Our toolbox integrates several open-source packages (ISCE, StaMPS & MintPy) to provide a coherent MTI processing chain and additional data management tools within an explicit and user-friendly graphical user interface. EZ-InSAR is not a ‘black box’; full and easy access to all the MTI processing parameters is provided for within the interface.

The EZ-InSAR MTI processing chain comprises three modules: (1) Data Preparation; (2) SAR Interferometry and (3) Time Series Analysis. In the first module, the only required inputs if processing Sentinel-1 data are a KML file delimiting the study area, a satellite path number, and a time span for analysis. The toolbox will then automatically download the Sentinel-1 SAR (with orbit files) and DEM data. In the second module, the user can coregister the SAR imagery (in SLC format) and generate interferograms. In the third module, either a Persistent Scatterers Interferometry (PSI) approach or a Short Baseline Subsets (SBAS) approach can be used to generate time series and velocity maps of ground displacement. Finally, EZ-InSAR will output the results of the MTI processing as surface velocity maps and time series data in GeoTiff, KML, and QGIS formats.

We illustrated the EZ-InSAR processing chain with Sentinel-1 IW data recently acquired over two ’restless’ volcanoes: Campi Flegrei Caldera, Italy; and Long Valley Caldera, USA. Both volcanoes have exhibited ground deformation in recent times, but they have contrasting land-cover characteristics, as well as different rates and temporal behaviour of ground motion. The results from EZ-InSAR show inflation at Campi Flegrei Caldera of up to 100 mm·yr^−1^ in 2021, whereas they reveal deflation at Long Valley caldera of up to -8 mm·yr^−1^ commencing in 2021. The spatial and temporal deformation pattern in the volcanic calderas as revealed by this MTI analysis agree reasonably well with local GNSS measurements at the two volcanoes.

In the future, we will continue develop the EZ-InSAR toolbox to enhance its functions. Improvements will include: (1) translation from MATLAB language to Python environment, (2) efficient updating ground displacements when a new SAR image acquisitions is added to the stack, (3) incorporating more recent and advanced MTI techniques (e.g., Distributed Scatters Interferometry), (4) more sophisticated visualisation and plotting tools, and (5) capacity to process free and open data from upcoming SAR satellite missions (e.g., Sentinel-1 C/D, Tandem-L and NiSAR).

We expect that the EZ-InSAR toolbox can serve as a valuable tool for both research and education. It provides enhanced capacity for citizens and professionals to monitor ground displacements and evaluate geohazards.

## Data Availability

Data are available on request from the authors.

## Availability and requirements

The source codes and the documentation are available via GitHub: https://github.com/alexisInSAR/EZ-InSAR. The program languages are MATLAB, Python3 and Bash shell. After installation of MATLAB (> 2020b), Python3, ISCE, StaMPS and MintPY software, the scripts have been developed and tested on Linux OS (Ubuntu 20.04). The full program size is around 15 MB (including 14 MB for documentation).
